# Supplementary material for: Evaluation of the impact of PEPFAR transition on retention in care in South Africa’s Western Cape
Source: medRxiv. 2023 Apr 14:2023.01.20.23284819. Originally published 2023 Jan 20. Preprint. [Version 2] doi: 10.1101/2023.01.20.23284819 (PMC9882633; doi:10.1101/2023.01.20.23284819)
Supplement: Supplement 1 [file media-1.docx]

**Supplemental Figure 2a-2e. 12 month retention in care, loss to follow-up and death stratified by NGO and overlaid on total patients on ART at the start and end of the 12-month period.**


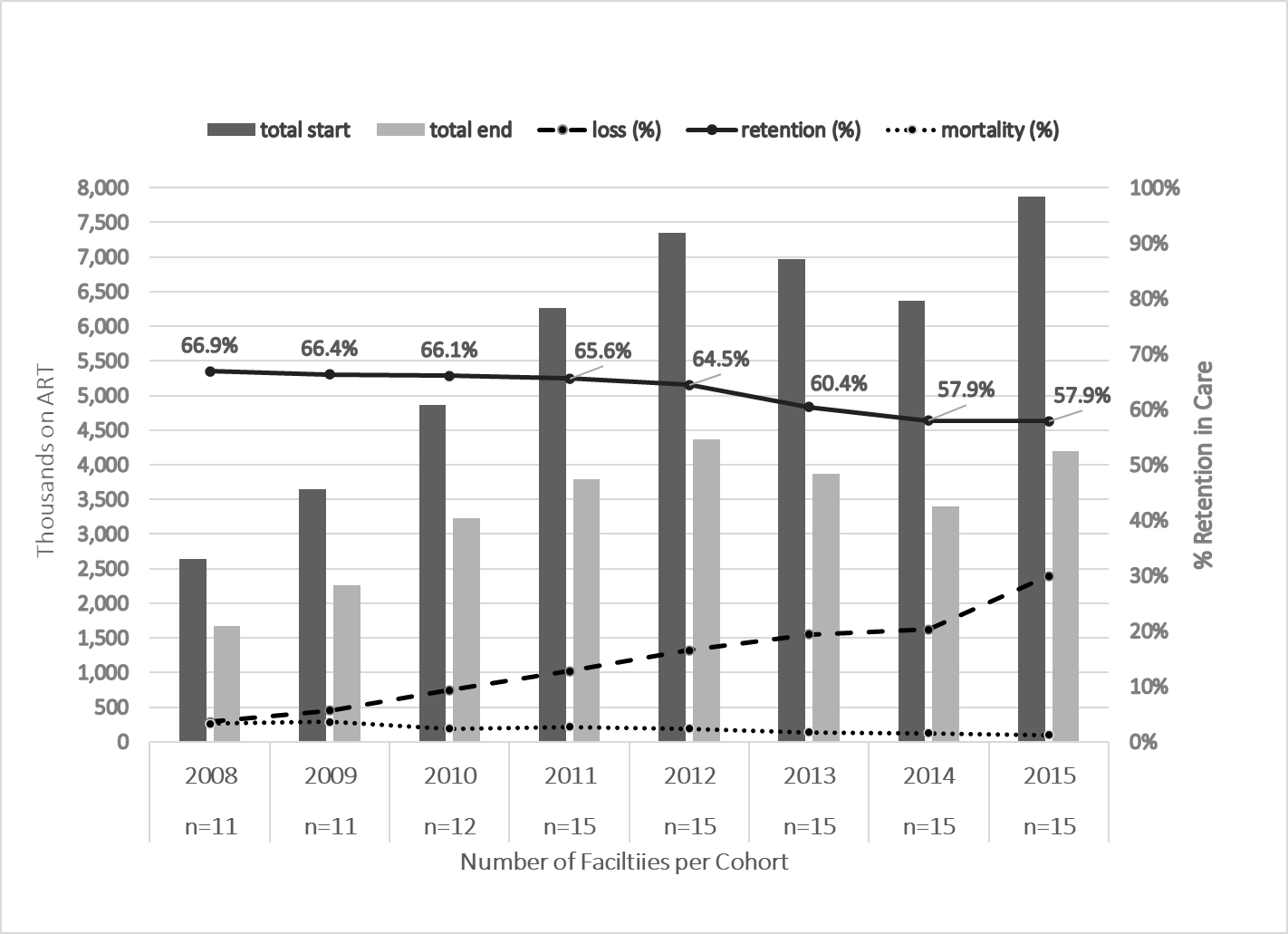


1. **Anova/Kheth’Impilo**

**d) Anova**

1. **that’sIt**

**b) Kheth’Impilo**

**
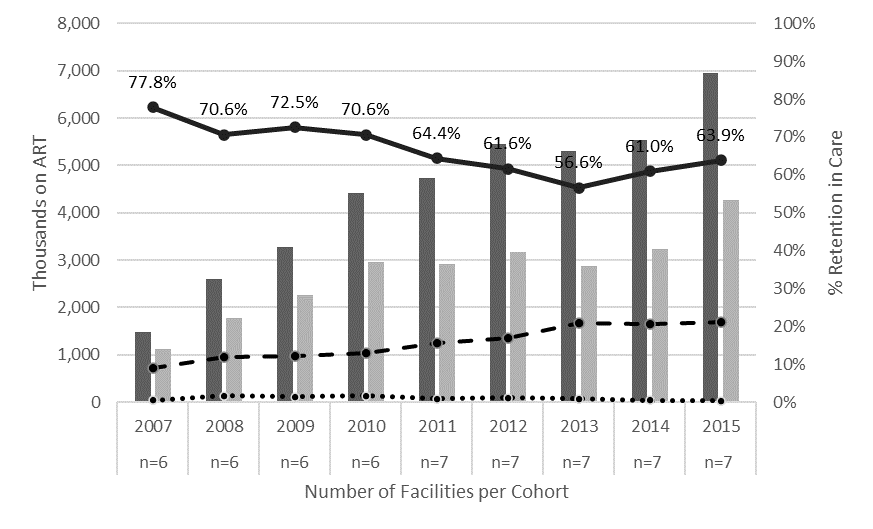

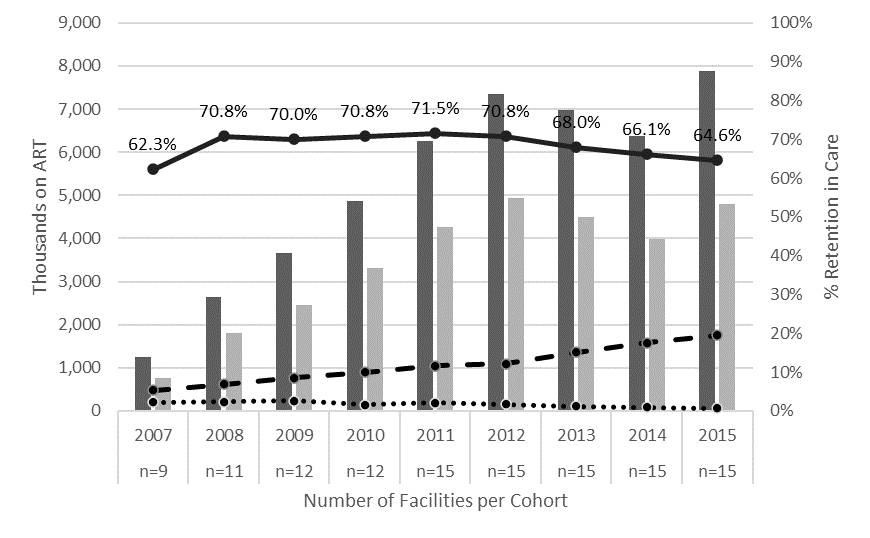
**

**
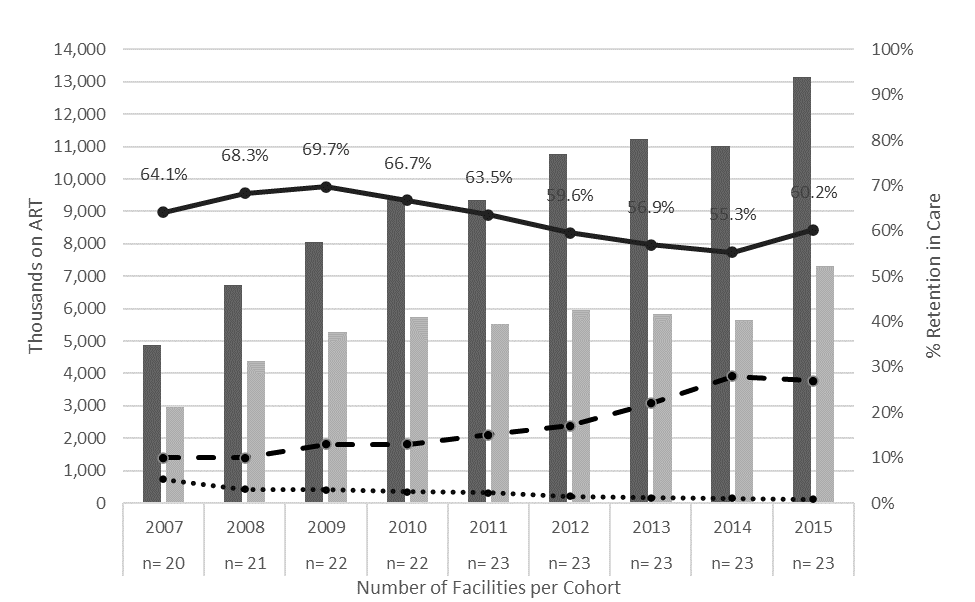

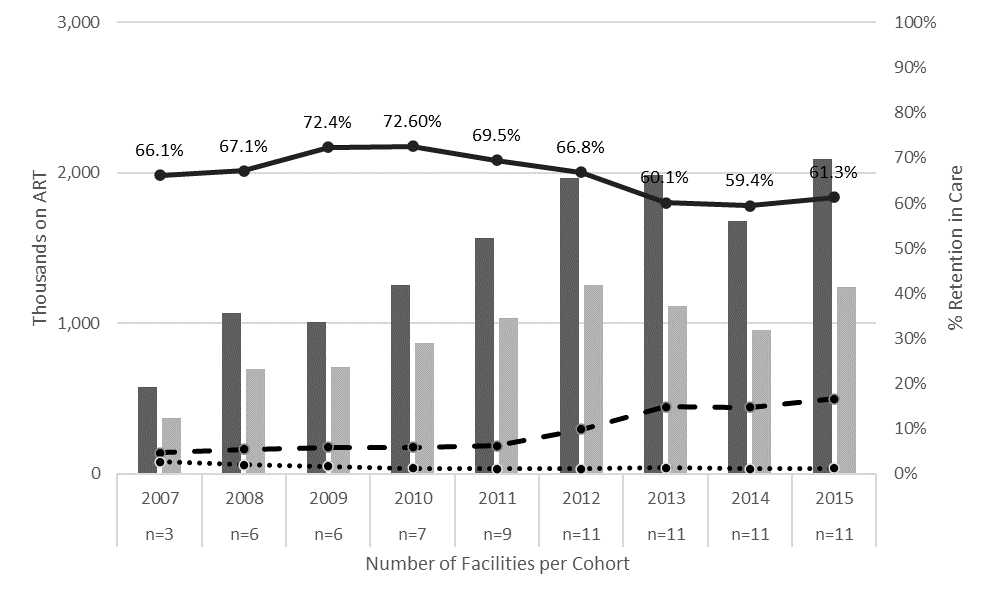
**

**E e) Right to Care**

**
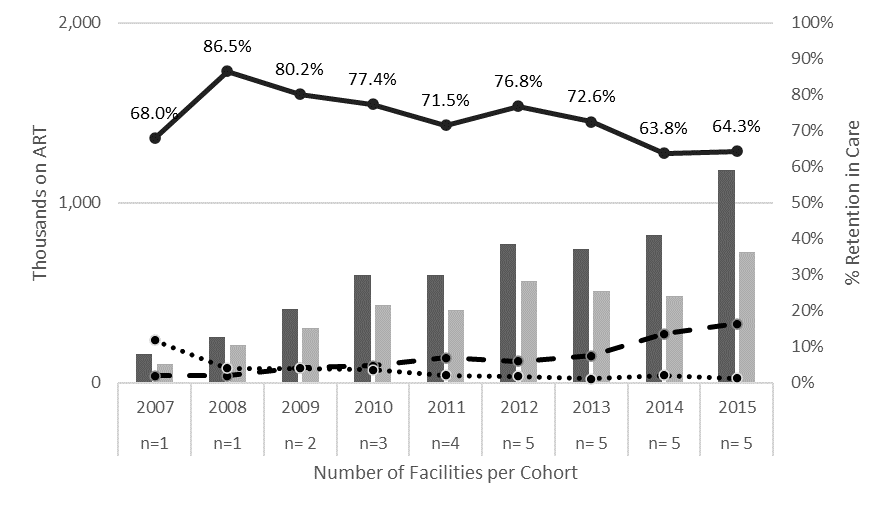
**
