# Supplementary figures and images for: Evaluation of the impact of PEPFAR transition on retention in care in South Africa’s Western Cape
Source: medRxiv. 2023 Apr 14:2023.01.20.23284819. Originally published 2023 Jan 20. Preprint. [Version 2] doi: 10.1101/2023.01.20.23284819 (PMC9882633; doi:10.1101/2023.01.20.23284819)

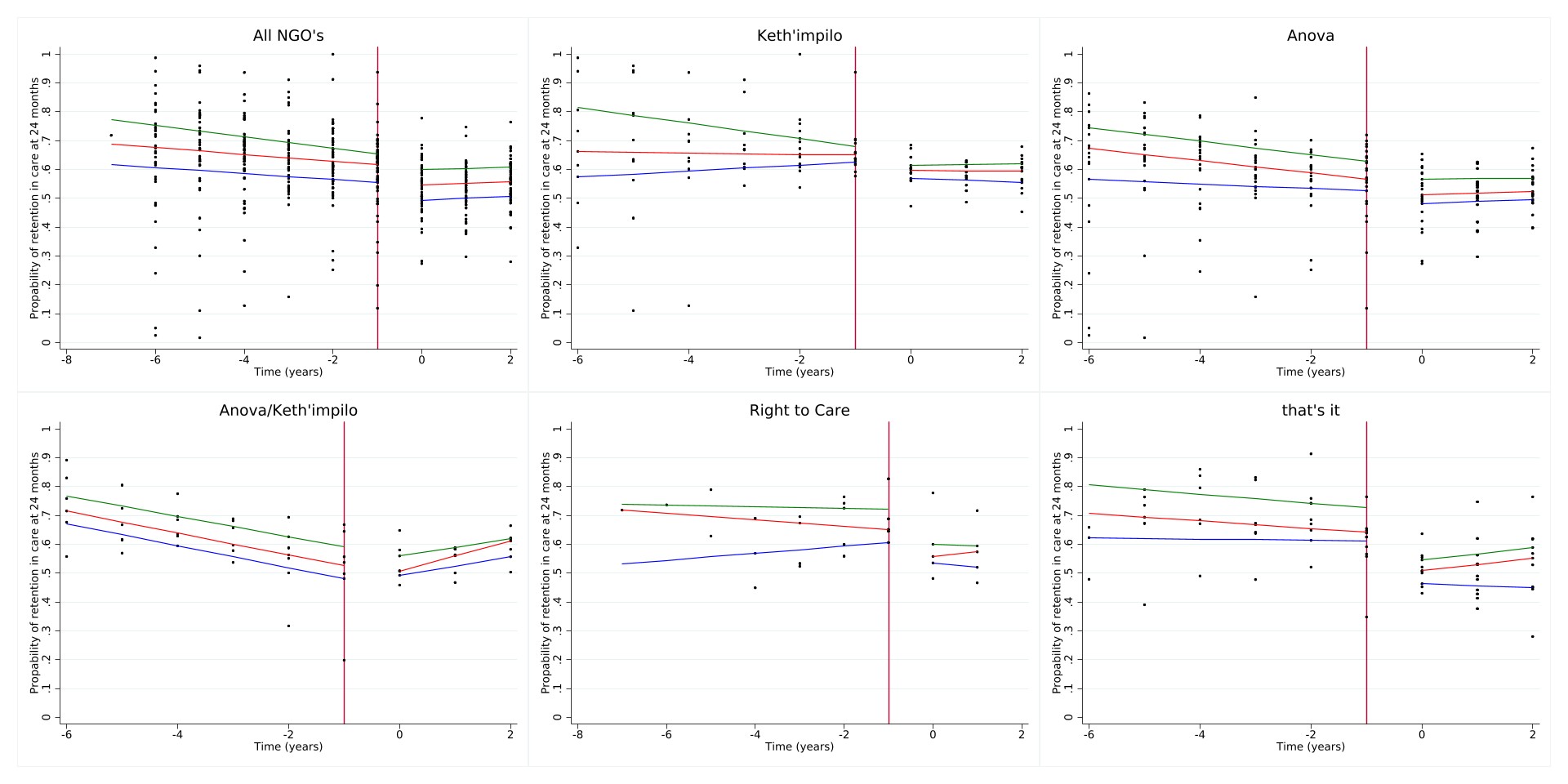
**Supplemental Figure 1a-1f. Quantile regression estimates for 24-month retention stratified by NGO**

Supplement: Supplement 4 [file media-4.docx]
